# How transformational leadership of managers affects employee innovative behavior in IT corporations

**DOI:** 10.3389/fpsyg.2025.1565307

**Published:** 2025-04-30

**Authors:** Jung Yon Kim, Dong-Yeol Yoon

**Affiliations:** ^1^SnC Management Consulting, Department of Human Resources, Seoul, Republic of Korea; ^2^Department of Business Administration, Konkuk University, Seoul, Republic of Korea

**Keywords:** transformational leadership, innovative behavior, psychological capital, thriving at work, IT company

## Abstract

Organizations are constantly challenged by new technologies that have the potential to transform their business models and organizational identity, and they are working to create an environment that supports innovation. Advances in digital tools for work have transformed the way we work, which used to be geographically constrained. Transformational leadership is becoming more important, facilitating access to information and knowledge sharing among internal constituents like never before. In addition, in innovation-oriented workplaces, frequent experiences of psychological capital and thriving at work, where employees enjoy an optimal state of challenge, are factors that promote innovative behavior. Based on the conservation of resources theory and social cognitive theory, this study explores the mechanisms through which transformational leadership influences the innovative behavior of IT workers. Specifically, it examines the dual mediating roles of psychological capital and thriving at work. Drawing on data from 394 valid responses out of 458 Korean IT workers surveyed, the results indicate that transformational leadership positively affects innovative behavior, with this relationship being partially mediated by employees’ psychological capital and thriving at work. These findings offer practical insights into how psychological capital and thriving at work function as critical psychological processes through which transformational leadership, within internal collaboration platforms, fosters innovative behavior among team members.

## 1 Introduction

The rise of various digital technologies is ushering in a new wave of disruption across industries, fundamentally reshaping traditional business strategies and processes. Additionally, economic and geographical uncertainties make it increasingly challenging to achieve profitability and growth without sustained innovation (Anser et al., 2021). In a market-driven economy, small and medium-sized enterprises face significant pressures to develop survival strategies that go beyond technology development. Most management policies in these enterprises demand agility and innovation, often relying on a limited pool of human capital (Benitez et al., 2018; Kim et al., 2022). Successful innovation, however, hinges on the willingness and capability of employees to innovate. These employees are vital to maintaining organizational stability while driving continuous innovation in a rapidly evolving environment (Grant, 2021).

Digital transformation trends necessitate new socialization processes within IT companies (Greimel et al., 2023; Sun et al., 2024). These processes enable leader-member interactions where leaders’ signals are clearly understood without the need for face-to-face communication, and diverse employee needs are addressed in real time. Within this framework of digital social influence, transformational leadership emerges as one of the most impactful leadership styles significantly driving innovation (Banks et al., 2022). Given the complexity of how individuals generate and implement ideas, numerous connections and influences exist between transformational leadership and individual innovative behavior. Consequently, there is an increasing need to explore the positive contextual conditions and psychological mechanisms through which transformational leaders promote employees’ innovative behavior (Lei et al., 2020).

Organizations have increasingly shifted their perspective, viewing their members not as passive individuals who simply address weaknesses or challenges, but as dynamic contributors capable of becoming proficient and efficient in alignment with their own preferences (Ullah et al., 2023). Employees are now recognized as enterprising intangible assets, able to recover from adversity without succumbing to despair (Gong and Yoon, 2021). In this context, psychological capital—a critical intangible asset that encapsulates the fundamental nature of human behavior—plays a pivotal role in predicting work-related outcomes (Hendriks et al., 2020). Understanding the causal links between developing psychological resources that positively influence employees is crucial, as these resources drive adaptability in unpredictable environments and significantly contribute to organizational innovation (Alshebami, 2021; Hsu and Chen, 2017; Amabile and Pratt, 2016).

In organizations, thriving at work represents a positive psychological state characterized by vitality and learning—a combination of feeling energized and passionate about one’s work and effectively applying self-acquired knowledge to tasks (Spreitzer et al., 2005). Thriving is influenced by individual characteristics and leadership, as employees with higher levels of prosperity are more likely to exhibit responsible behaviors that drive constructive change (Frazier and Tupper, 2018). Specifically, according to the socially embedded model of prosperity, transformational leadership positively impacts thriving at work by actively fostering members’ success and development. This enhanced state of thriving subsequently contributes to improved performance and innovation (Gerbasi et al., 2015; Kleine et al., 2019; Porath et al., 2012).

This study aims to provide deeper insights into the underlying psychological mechanisms that sequentially mediate the relationship between transformational leadership and employees’ innovative behavior. It examines these mechanisms within the context of small and medium-sized enterprises in the IT industry that utilize digital tools, drawing on the frameworks of conservation of resources theory and social cognitive theory.

## 2 Theory and hypotheses

### 2.1 Relationship between transformational leadership and employees’ innovative behavior

While IT companies have traditionally established themselves as service providers leveraging IT information, they have recently evolved into business model architects, delivering marketable product solutions that customers value (Gupta and Bose, 2022; Trabucchi and Buganza, 2020). This shift requires IT companies to reorganize their digital technologies, processes, and resources into innovative business models that meet market demands. Moreover, the IT industry consists of a spatially agnostic, project-based workforce of experts, which must maximize its members’ value through an augmented and connected workforce (Moencks et al., 2022; Leonardi, 2017; Khan et al., 2024). To achieve this, leaders must cultivate innovation by fostering a shared mindset and a supportive cultural attitude.

Innovative behavior refers to the process by which employees generate and act on new ideas that contribute to organizational performance (Sanz-Valle and Jiménez-Jiménez, 2018). It encompasses not only the creation of novel and useful ideas but also their deliberate implementation. Research suggests that employee innovative behavior is driven by three key antecedents: innovative cognition, interaction, and an innovation-friendly climate (AlEssa and Durugbo, 2022).

Conversely, the various physical constraints imposed by COVID-19 have forced organizations to overcome leader-member connectivity challenges through remote work and virtual communication. Organizations are investing in digital tools to enhance connectivity and collaboration among members, thereby driving innovation (Cai et al., 2018). Digital tools, such as Zoom and Google Workspace, foster environments that enable active participation in work-related social interactions (Leonardi, 2017). This new way of working necessitates a leadership style where leaders provide clear direction and decision-making to drive change, encourage followers to express ideas, and facilitate the sharing of work-related information (Khan et al., 2024; Opland et al., 2022). Transformational leadership has thus become essential for tangibly communicating clear expectations and goals throughout interactions, while effectively addressing current organizational challenges (Grošelj et al., 2020).

According to the conservation of resources theory, social and cognitive interactions between leaders and members enhance performance by strengthening individuals’ psychological resources and values (Krishen et al., 2016; Hobfoll et al., 2018). Transformational leaders serve as a vital resource by understanding, recognizing, and supporting employees’ ideas, fostering an environment where employees feel equipped to handle their work (Bakker and Demerouti, 2017; Eva et al., 2019). In this context, leaders view employees as valuable human capital, provide individualized feedback, and nurture future achievement and job value through supportive relationships. This emotional support enhances employees’ mental resilience, enabling them to generate new ideas even in the face of failure during challenging tasks, thereby sustaining their innovative behavior (Afsar et al., 2019).

However, the visibility of work enabled by the implementation of new technologies minimizes duplication, allows members to freely utilize and share specialized knowledge based on their intentions and goals, and fosters a strong sense of community within the organization through active participation (Ng and Yee, 2020). This visibility empowers individuals with the confidence to effectively fulfill their roles and responsibilities while carefully evaluating and addressing the potential outcomes of utilizing their competencies.

According to social cognitive theory, individuals rely not only on their cognitive to learn and maintain behavioral patterns but also on social relationships and environmental influences to shape their behavior (Kwahk et al., 2018; Afsar and Umrani, 2020). Specifically, the advancement of IT technology encourages active participation by enabling members to share their opinions on relevant issues through collaboration platforms such as Microsoft Teams and Slack, exchange knowledge and experiences with peers or leaders, and accelerate the socialization process. This process fosters a perception of work as meaningful, ultimately leading to a set of innovative behavior (Leonardi, 2017; Xu and Suntrayuth, 2022).

These concepts lead to the following hypothesis:

*H1:* Transformational leadership positively influences innovative behavior.

### 2.2 Relationships among transformational leadership, employees’ psychological capital, and employees’ innovative behavior

Psychological capital represents a comprehensive source of energy, encompassing positive psychological states such as self-efficacy, hope, optimism, and resilience. It has a broad influence on members’ attitudes and behaviors, particularly as shaped by leaders (Luthans et al., 2013). Ahmed et al. (2018) highlighted the importance of leaders stimulating employees’ psychological states to foster innovative behavior.

In the knowledge-intensive IT industry, employees face numerous opportunities owing to evolving job demands and the need to respond rapidly to change (Khan et al., 2024). IT professionals need to continuously acquire new skills to keep up with emerging technologies such as AI and Cloud. While this presents the challenge of learning new tools and programming languages, it also offers an opportunity to enhance their expertise and remain competitive. Consequently, leaders must implement effective project management strategies that align with the unique characteristics of each project while providing the necessary resources to foster active employee participation.

Demerouti and Bakker (2011) argued that employees perceive organizational support, autonomy, and feedback from supervisors as valuable resources they can draw upon. Leaders’ intellectual stimulation related to work is viewed by employees as a positive cognitive resource that helps them maintain, develop, and invest their personal resources. Employees often rely on information from their supervisors and close peers within the organization to build confidence in their ability to succeed at challenging tasks (Sun et al., 2024). In IT organizations, where digital tools are frequently utilized, leaders can offer constructive feedback and access to critical information to help employees develop self-efficacy even when they encounter obstacles or make mistakes. Transformational leaders play a key role in stimulating energy and motivation for achievement, fostering a dynamic process that builds confidence in new approaches despite risks and challenges (Grant and Berry, 2011; Yin et al., 2020). Self-efficacy, a core component of psychological capital, is a vital asset for individuals in organizations as it enables effective communication and problem-solving. As most ideas stem from generalized beliefs, self-efficacy serves as a crucial foundation for materializing these ideas (Ng and Lucianetti, 2016).

The verbal persuasion and confidence conveyed by transformational leaders instill in employees a belief in their abilities and optimism for the future (Gooty et al., 2009; Rego et al., 2019). Hope, as a component of psychological capital, is a critical future-oriented psychological resource that shapes individuals’ attitudes and behaviors, enabling them to proactively address difficulties and challenges (Snyder, 2002; Ullah et al., 2023).

Hope establishes a goal-oriented pathway and provides the momentum to transform challenges into opportunities, fostering the ability to generate ideas from a fresh perspective. Individuals with higher levels of hope are more skilled at setting challenging goals and identifying the resources necessary to achieve them. Consequently, even in the face of obstacles, they maintain a positive outlook and continually strive to exhibit innovative behavior (Luthans et al., 2008).

The individualized consideration and intellectual stimulation provided by transformational leadership influence how employees perceive work-related stress, fostering resilience to manage demanding job requirements (Hentrich et al., 2017). Resilience, as a component of psychological capital, refers to an individual’s ability to gather, select, and utilize resources to effectively respond to psychological stress. It represents the personal capacity to identify and leverage both internal and external resources to overcome adversity or restore balance after experiencing setbacks (King et al., 2016). Transformational leaders can help reduce employees’ stress by offering positive alternatives and resources that enable them to explore new approaches to problem-solving (Diebig et al., 2016).

According to social cognitive theory, vicarious experiences through role modeling enhance self-efficacy. Leaders with high self-efficacy instill in employees the belief that they play a pivotal role in the organization, as employees admire and identify with such leaders (Nolzen, 2018). Beyond individual project experience, the success of an IT company relies on collaboration and connection among diverse groups, fostering a sense of meaning and active motivation to work (Khan et al., 2024; Ren and Sun, 2023).

Highly intellectualized employees are motivated to share resources, build relationships, and communicate, particularly when these actions align with their personal values or criteria. This active motivation is driven by perceptions of self-efficacy, feelings of control, reasons and beliefs that sustain persistent behavior, and the cognitive processes involved in actively setting goals (Tams et al., 2018). Consequently, an individual’s cognitive processes, social learning experiences, self-efficacy, and beliefs interact to shape psychological capital, which, in turn, fosters innovative behavior.

Transformational leaders enhance employees’ sense of control and the meaningfulness of their work by fostering a positive self-concept, motivating them, and helping them recognize the importance and impact of their efforts on organizational innovation (Tse et al., 2018; Scott and Bruce, 1994; Han et al., 2016). When employees attribute meaningfulness to their actions, they actively pursue goals, develop innovative ideas, and generate alternative pathways to achieve those goals.

This highlights that individuals with high levels of hope possess the cognitive capacity for self-regulation, which equips them with the initiative, self-control, and proactivity required to accomplish their objectives (Rego et al., 2019; Ullah et al., 2023).

Highly resilient individuals can adapt to change, develop new strategies for coping with uncertainty, and recover from adverse emotional experiences by fostering an atmosphere that promotes psychological safety for themselves and others (Hendriks et al., 2020). Resilience, as a component of psychological capital, supports adaptability and flexibility in uncertain situations. An optimistic outlook further cultivates an environment that encourages openness and experimentation.

This positive psychological capital empowers individuals to exhibit innovative behavior by encouraging them to explore alternatives in the face of initial setbacks and failures. It also strengthens their willpower and determination to achieve their goals (Youssef and Luthans, 2007). Individuals with high psychological capital in the IT industry generally adopt a creative approach to attaining their goals (Ullah et al., 2023). They often make voluntary efforts to create, promote, and implement innovative behavior in their work environment. These qualities manifest as creative thinking and proactive personality traits, serving as a driving force behind positive change within an organization.

These concepts lead to the following hypothesis:

*H2:* Psychological capital mediates the relationship between transformational leadership and innovative behavior.

### 2.3 Relationships among transformational leadership, employees’ thriving at work, and employees’ innovative behavior

IT jobs foster job engagement by requiring complex problem-solving processes that involve transforming ideas into technology (Su et al., 2018). Thriving at work is a self-adaptive process where individuals recognize the direction of their work, develop new skills, and actively pursue opportunities (Spreitzer et al., 2012).

While thriving at work may sometimes stem from the intrinsic enjoyment of the activity itself, it is often driven by learning goals aimed at achieving personally meaningful accomplishments and recognition (Sonenshein et al., 2013). Thriving at work, nurtured by social interactions between leaders and employees, plays a critical role in encouraging employees to think and act creatively beyond their typical roles and responsibilities (Guan and Frenkel, 2020).

In the socially embedded model, thriving at work is driven by the acquisition of positive meanings and affective resources, which enable individuals to pursue innovative behavior (Carmeli and Spreitzer, 2009; Guan and Frenkel, 2020). This suggests that contextual resources are a critical factor in fostering thriving at work. Kleine et al. (2019) argued that contextual resources, such as social support from family, spouses, and supervisors, enhance individuals’ thriving at work. Similarly, Yin et al. (2020) highlighted that transformational leadership, as a contextual resource, promotes learning and vitality by providing personalized support, showing interest in employees’ growth, and offering continuous feedback on challenging tasks. Transformational leadership also fosters respect and enthusiasm, encouraging members to experience positive reinforcement, well-being, and openness within the organization (Hildenbrand et al., 2018; Zia et al., 2022).

In IT organizations, leaders influence employees’ vitality by increasing the availability of resources needed to accomplish their work (Luqman et al., 2021; Men, 2014). Transformational leaders prioritize employees’ interests, facilitate information sharing, and provide rich relational resources, enabling employees to gain additional knowledge. Moreover, the experience of positive emotions builds psychological and social resources, empowering individuals to engage in innovative behavior (Fredrickson et al., 2008; Wallace et al., 2016).

Employees with high levels of thriving at work demonstrate a strong desire to acquire and disseminate new knowledge and skills within the organization. They also dedicate significant energy to applying these skills in practice, which fosters innovation (Guan and Frenkel, 2020). Thriving employees enjoy independent thinking, and the positive emotional resources they develop encourage them to seek ways to influence others and become more actively involved in innovative activities.

In social cognitive theory, it is argued that transformational leadership in IT corporations influences thriving at work by reflecting a human agency perspective, wherein employees actively participate in their own success and development (Tintoré, 2019; Graziotin et al., 2018). This perspective emphasizes that leaders foster quality relationships to achieve common goals, while employees leverage opportunities to engage in three proactive work behaviors: task focus, heedful relating, and exploration.

Employees who value agency believe that they can exert significant control over their work and act responsibly to achieve outcomes they find meaningful (Goh et al., 2022; Wallace et al., 2016).

IT skills are developed through knowledge sharing and collaboration among team members. Employees’ role expectations and their positive perceptions of their leaders reinforce certain behaviors that contribute to the intrinsic enjoyment of work (Zubair and Kamal, 2015; Su et al., 2018). Self-regulated behaviors, such as taking on challenging tasks, responding promptly to feedback, and avoiding harmful actions, are critical in this context. These behaviors influence innovative behavior through positive motivational processes, helping employees recognize the significance of their work, the strength of their energy, and the applicability of their learning (Kleine et al., 2019; Niessen et al., 2012).

As a result, employees invest greater effort and energy in innovative behavior—not only to generate new ideas but also to implement them within the organization. When employees perceive that the organization is focused on learning and improvement, they are motivated to identify problems and propose solutions, which fosters innovative behavior.

These concepts lead to the following hypothesis:

*H3:* Employees’ thriving at work mediates the relationship between transformational leadership and innovative behavior.

### 2.4 The dual mediating effect of psychological capital and thriving at work

Paterson et al. (2014) demonstrated that psychological capital enhances employees’ work performance through a virtuous cycle of resource acquisition and utilization. This implies that an individual’s ability to acquire resources keeps them competitive, while the constructive interaction of resources within the organization plays a crucial role in driving performance. Transformational leadership acts as a catalyst for building new types of relationships, enabling employees to perform their tasks effectively and providing the resources needed to work confidently beyond the expectations of explicit exchange contracts (Lei et al., 2020; Dvir et al., 2002). This dynamic creates a beneficial cycle in which employees view their leader’s goals and direction as meaningful resources that align with their work. This perception fosters hope and confidence in the future, encouraging the reinvestment of time and energy, which ultimately cultivates a sense of prosperity (Grover et al., 2018).

Thriving at work is not a static state but a dynamic process, representing continuous change and development for individuals (Hyde et al., 2022). This suggests that an individual’s emotional and cognitive thriving at work within an organization can be significantly influenced by environmental factors. When employees perceive that their leaders foster a resource-rich work environment, it enhances their psychological capital and thriving at work, leading to a repertoire of thoughts and behaviors that support innovative actions (Shahid and Muchiri, 2019; Goh et al., 2022; Christensen-Salem et al., 2021).

Transformational leaders play a crucial role in helping employees maintain stable psychological capital by fostering positive work motivation and perseverance, which in turn drives innovation through thriving at work. Additionally, individualized care, such as mentoring from leaders, provides employees with emotional resources to mitigate the depletion of psychological resources caused by work stress (Rego et al., 2019; Allen et al., 2017; Dvir et al., 2002; Bednall et al., 2018).

Leaders in IT organizations motivate employees by providing visibility and clarity in communication (Men, 2014). This ensures that the leader’s vision is effectively conveyed and easily observable. According to social cognitive theory, anticipated expectations align with employees’ individual thoughts and beliefs, while connectivity through interaction satisfies psychological needs, fostering a sense of emotional security about the future (Khan, 2023; Bastardoz and Van Vugt, 2019).

Emotional safety does not imply isolation but rather feeling respected for one’s contributions as part of a team. The psychological experience of being valued for one’s passion and dedication enhances employees’ vitality. This, in turn, fosters innovative behavior by enabling individuals to learn from the observed perspectives and experiences of others.

Transformational leadership fosters innovative behavior through cognitive processes by influencing employees’ beliefs, accountability, and goals (Kuo et al., 2021; Ng and Lucianetti, 2016). Psychological capital, a relatively malleable resource, can be enhanced through appropriate interventions, while the thriving at work driven by subjective experiences contributes to innovation over time (Hyde et al., 2022; Grover et al., 2018).

Transformational leadership instills confidence in employees’ abilities and promotes self-regulation, enabling them to recover and persevere when facing challenges (Schornick et al., 2023; Linnenluecke, 2017). Furthermore, when individuals experience learning within an organization, they are motivated to sustain and expand that sense of growth by engaging in developmental activities, regardless of intent or timing (Gao and Wu, 2020; Hsu and Chen, 2017). This indicates that as individuals perceive themselves to be progressing, they actively seek opportunities to acquire new knowledge and skills to advance their careers, ultimately driving innovative behavior.

In this context, we propose the following hypothesis:

*H4:* Employees’ psychological capital and thriving at work sequentially mediates the relationship between transformational leadership and innovative behavior.

## 3 Research method

**Figure 1:**
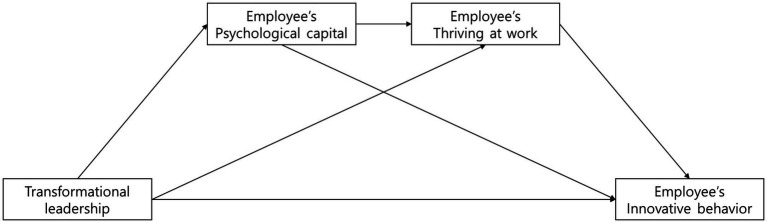
Hypothesized model.

The hypothesized framework guiding this study is depicted in Figure 1.

### 3.1 Participants and procedure

To test the research hypothesis, this study was conducted in two phases: a preliminary survey and a main survey, targeting employees in small and medium-sized enterprises (SMEs) within the IT sector. SMEs are more likely to be influenced by intangible assets, such as psychological capital, due to their limited resources. In contrast, large enterprises may exhibit different mechanisms that influence innovative behavior, driven by their complex hierarchical structures and formalized systems. The preliminary survey was administered both online and offline from February 13 to 20, 2024, to employees working in development departments or research labs within IT SMEs.

The main survey focused on employees who use workplace collaboration tools within their organizations to enhance community connectivity and visibility with their leaders. Employees familiar with these platforms contribute to improved productivity and organizational performance by fostering agile collaboration, knowledge sharing, organizational learning, and innovation (Alimam et al., 2017).

A total of 87 responses were received for the preliminary survey, of which 83 were analyzed after excluding incomplete submissions. The preliminary survey was revised and supplemented to address questions that compromised reliability and validity.

The online questionnaire for the main survey was distributed from February 28, 2024, to March 11, 2024. Out of 458 responses collected, 394 were used for analysis after excluding incomplete or invalid responses.

### 3.2 Measures

Transformational leadership is defined as leadership that encourages employees to explore new ways of working or to be proactive in their tasks by setting challenging goals and instilling confidence in their ability to achieve them (Zia et al., 2022). In this study, 12 items developed by Bass and Avolio (1994) were translated into Korean to measure transformational leadership, using a 5-point Likert scale (1 = not at all, 5 = very much). Cronbach’s alpha was 0.913.

Psychological capital is defined as a psychological state encompassing an individual’s confidence in completing challenging tasks, belief in present and future success, determination to achieve goals, and resilience in the face of challenges (Luthans et al., 2007). A 12-item questionnaire adapted from the shortened version of Luthans et al.’s (2007) original 24-item scale was used to measure psychological capital on a 5-point Likert scale. It has the advantage of reducing participant fatigue within a short period of time, while its items are easy to translate and apply across various cultures. Cronbach’s alpha was 0.853.

Based on Porath et al. (2012), thriving at work is defined as a state in which individuals experience and exert both mental, physical, and social vitality and full learning possible in their daily lives. The 10 original scales of thriving at work developed by Porath et al. (2012) were validated among Koreans, and eight of the 10 scales were back-translated to non-negatively worded items, and the items were then utilized to create a Likert 5-point scale. Lee and Lee (2021) demonstrated that the eight items, excluding the reverse-coded ones, exhibit excellent discriminant validity, convergent validity, and reliability for the Korean context. Cronbach’s alpha was 0.887.

Innovative behavior is defined as a set of activities that includes generating new and useful ideas, gaining support for those ideas, and converting them into actionable outcomes (Wang, 2021; Janssen, 2000). A 9-item questionnaire adapted from Janssen’s (2000) study was used to measure innovative behavior on a 5-point Likert scale. Cronbach’s alpha was 0.858.

This study controlled for demographic variables such as gender, age, education, job group, position, tenure, and use of collaboration tools at work, as these factors are thought to influence psychological capital, thriving at work, and innovative behavior (Sun et al., 2024; Benitez et al., 2018; Grover et al., 2018; Kleine et al., 2019). Gender, age, and job group were treated as dummy variables in the analysis. Generational differences among employees were also considered, as factors like work-life balance, the value and meaning of work, career patterns, and leadership preferences may influence attitudes and innovative behavior.

The MZ generation, with their digital fluency and collaborative problem-solving skills using IT tools, drives innovation through their adaptability to change and preference for horizontal structures (Zia et al., 2024; Seo, 2024). Their innovative potential within organizations continues to strengthen as digital technology and collaboration tools proliferate, enhancing their distinctive capabilities.

## 4 Results

### 4.1 Characteristics of the sample

A frequency analysis was conducted to examine the demographic characteristics of the survey respondents. Out of a total of 394 respondents, 274 (69.5%) were male, and 120 (30.5%) were female. Regarding age, 245 respondents (62.2%) belonged to the MZ generation as of the survey date, while 149 respondents (37.8%) were from Generation X. In terms of education, 263 respondents (66.8%) held a bachelor’s degree, followed by 80 (20.3%) with a master’s degree. By position, 102 respondents (25.9%) were managers, followed by 78 (19.8%) assistant managers, and 75 (19.0%) deputy managers. Regarding tenure, 115 respondents (28.8%) had over 13 years of experience, followed by 67 (17.0%) with 2 to 5 years, and 65 (16.5%) with 5 to 8 years. For experience with enterprise social media usage, 147 respondents (37.3%) reported using such platforms for 1 to 3 years, followed by 93 (23.6%) who had used them for less than 1 year and 83 (21.1%) who had more than 5 years of experience.

### 4.2 Correlation analysis

Pearson’s correlation analysis was conducted to examine the relationships between the main variables in the research model and the control variables. The correlation coefficient (*r*) was interpreted as follows: values closer to 1 indicate a stronger positive correlation, while values closer to 0 indicate a weaker correlation.

As shown in Table 1, transformational leadership was significantly correlated with age (*r* = −0.116, *p* < 0.05), position (*r* = 0.119, *p* < 0.05), and tenure (*r* = 0.113, *p* < 0.05). Psychological capital was significantly correlated with age (*r* = −0.122, *p* < 0.05), education (*r* = 0.219, *p* < 0.001), position (*r* = 0.155, *p* < 0.01), and transformational leadership (*r* = 0.310, *p* < 0.001). Thriving at work was significantly correlated with age (*r* = −0.104, *p* < 0.05), education (*r* = 0.133, *p* < 0.01), job management (*r* = 0.105, *p* < 0.05), position (*r* = 0.110, *p* < 0.05), transformational leadership (*r* = 0.386, *p* < 0.001), and psychological capital (*r* = 0.749, *p* < 0.001). Innovative behavior was significantly related to gender (*r* = 0.114, *p* < 0.05), age (*r* = −0.180, *p* < 0.001), education (*r* = 0.185, *p* < 0.001), position (*r* = 0.244, *p* < 0.001), tenure (*r* = 0.198, *p* < 0.001), transformational leadership (*r* = 0.363, *p* < 0.001), employee psychological capital (*r* = 0.605, *p* < 0.001), and thriving at work (*r* = 0.555, *p* < 0.001).

**Table 1:** Correlation between variables.

Variables	M	SE	1	2	3	4	5	6	7	8	9	10	11	12
1. Gender	0.70	0.46	1											
2. Age	0.62	0.49	−0.289^***^	1										
3. Education	3.16	0.72	0.052	−0.090	1									
4. Job Management	0.10	0.31	0.045	−0.128^*^	−0.043	1								
5. Job R&D	0.71	0.45	−0.082	0.091	0.263^***^	−0.534^***^	1							
6. Position	4.01	1.57	0.377^***^	−0.567^***^	0.295^***^	0.062	0.077	1						
7. Tenure	3.96	1.73	0.327^***^	−0.497^***^	0.180^***^	0.069	0.000	0.687^***^	1					
8. ESM	3.37	1.06	0.041	−0.062	0.004	0.016	0.019	0.180^***^	0.277^***^	1				
9. Transformational Leadership	3.23	0.67	0.096	−0.116^*^	0.051	0.048	0.016	0.119^*^	0.113^*^	0.029	1			
10. Psychological capital	3.66	0.52	0.032	−0.122^*^	0.219^***^	0.037	0.019	0.155^**^	0.089	0.073	0.310^***^	1		
11. Thriving at work	3.43	0.68	0.083	−0.104^*^	0.133^**^	0.105^*^	0.001	0.110^*^	0.080	0.093	0.386^***^	0.749^***^	1	
12. Innovative behavior	3.56	0.57	0.114^*^	−0.180^***^	0.185^***^	0.051	−0.058	0.244^***^	0.198^***^	0.096	0.363^***^	0.605^***^	0.555^***^	1

*n* = 394, ^*^p < 0.05, ^**^p < 0.01, ^***^p < 0.001.

Gender (Dummy variable): 1 = Male, 0 = Female / Age (MZ generation dummy variable): 1 = 18 years old to 43 years old, 0 = 44 years old to 63 years old / Education: 1 = High School, 2 = college graduate, 3 = bachelor’s degree, 4 = Master’s degree, 5 = Doctoral degree / Job management (Dummy variable): 1 = Management Support, 0 = Production, 0 = Research & Development, 0 = Sales/Marketing/Service, 0 = Other 5 / Job R&D (Dummy variable): 0 = Management Support, 0 = Production, 1 = R&D, 0 = Sales/Marketing/Service, 0 = Other 5 / Position: 1 = Staff, 2 = Administrative manager, 3 = Assistant Manager, 4 = Manager, 5 = Deputy Manager, 6 = Director, 7 = Executive / Tenure: 1 = Less than 2 years, 2 = More than 2 years to less than 5 years, 3 = More than 5 years to less than 8 years, 4 = More than 8 years to less than 10 years, 5 = More than 10 years to less than 13 years, 6 = More than 13 years / ESM (enterprise social media usage duration): 1 = No experience, 2 = Less than 1 year, 3 = More than 1 year to less than 3 years, 4 = More than 3 years to less than 5 years, 5 = More than 5 years.

### 4.3 Hypothesis results

The regression analysis results are summarized in Table 2, with gender, age, job type, position, education, tenure, and enterprise social media (ESM) usage included as control variables that may influence the main variables.

**Table 2:** Regression results.

Variables	Psychological capital		Thriving at work		Innovative behavior					
	Model 1	Model 2	Model 3	Model 4	Model 5	Model 6	Model 7	Model 8	Model 9	Model 10
Gender	−0.021	−0.038	0.059	0.037	0.017	−0.003	0.029	0.017	−0.014	−0.020
Age (MZ Gen)	−0.081	−0.063	−0.071^*^	−0.048	−0.057	−0.036	−0.010	−0.003	−0.019	−0.013
Education	0.206^***^	0.200^***^	0.120^*^	0.114^*^	0.163^**^	0.157^**^	0.045	0.053	0.099^*^	0.104
Job management	0.019	0.001	0.122	0.098	−0.026	−0.047	−0.037	−0.048	−0.090	−0.093
Job R&D	−0.027	−0.043	0.043	0.023	−0.120^*^	−0.139^*^	−0.105^*^	−0.116^*^	−0.143^**^	−0.149^**^
Position	0.087	0.082	0.010	0.004	0.142	0.136	0.092	0.094	0.137^*^	0.134^*^
Tenure	−0.062	−0.074	−0.037	−0.052	0.022	0.008	0.057	0.046	0.041	0.032
ESM	0.070	0.069	0.091	0.089	0.063	0.061	0.022	0.025	0.014	0.019
Transformational leadership		0.294^***^		0.369^***^		0.284^***^		0.185^***^		0.165^***^
Psychological capital							575^***^	0.518^***^		
Thriving at work									530^***^	0.468^***^
R^2^	0.068	0.152	0.048	0.181	0.093	0.204	0.402	0.432	0.361	0.384
Adj R^2^	0.048	0.132	0.028	0.161	0.074	0.185	0.388	0.417	0.346	0.368
△ R^2^		0.084		0.133		0.111	0.309	0.339	0.268	0.291
F For R^2^	3.486^**^	38.141^***^	2.414^**^	62.248^***^	4.939^***^	53.569^***^	198.032^***^	114.283^***^	160.092^***^	90.307^***^
Overall F	3.486^**^	7.636^***^	2.414^**^	9.404^***^	4.939^***^	12.663^***^	28.641^***^	29.133^***^	24.100^***^	25.463^***^

*n = 394, ^*^p < 0.05, ^**^p < 0.01, ^***^p < 0.001, Standardized Beta.

To test H1, Model 6 in Table 2 shows that transformational leadership has a significant positive effect on innovative behavior (*β* = 0.284, *p* < 0.001). The increase in explanatory power is statistically significant (ΔR^2^ = 0.204, F for ΔR^2^ = 53.569, *p* < 0.001). These results support H1, which states that transformational leadership positively impacts innovative behavior.

A three-step mediation analysis method (Baron and Kenny, 1986) was employed to test H2 and H3. First, to test H2, Model 2 shows that transformational leadership significantly and positively predicts employees’ psychological capital (*β* = 0.294, *p* < 0.001). Furthermore, as shown in Model 7, psychological capital significantly affects innovative behavior (*β* = 0.575, *p* < 0.001).

Model 8 demonstrates the mediating effect of psychological capital on innovative behavior (*β* = 0.518, *p* < 0.001). Notably, when the mediator is included, the *β* value for the independent variable decreases. Therefore, H2 is supported as a partial mediation effect.

To test H3, Model 4 shows that transformational leadership has a significant and positive predictive effect on employees’ thriving at work (*β* = 0.369, *p* < 0.001). Furthermore, as shown in Model 9, thriving at work significantly affects innovative behavior (*β* = 0.530, *p* < 0.001). Model 10 demonstrates the mediating effect of thriving at work on innovative behavior (*β* = 0.468, *p* < 0.001). Notably, when the mediator is included, the *β* value for the independent variable decreases. Therefore, H3 is supported as a partial mediation effect.

To examine the sequential mediation between employees’ psychological capital and thriving at work, Hayes’ (2017) Process Macro Model 6 was utilized. The significance of the serial mediation effect was further tested using SPSS Process Macro 4.2 with the bootstrap method and 95% confidence intervals (Preacher and Hayes, 2008).

Table 3 presents the indirect effects of the continuous mediation model on the relationship between transformational leadership and innovative behavior. The indirect effect of transformational leadership → employees’ psychological capital → innovative behavior was significant at the 95% confidence level, with a value of *β* = 0.0959 (LLCI = 0.0525, ULCI = 0.1497). As the confidence interval does not include zero, this effect is considered statistically significant. Similarly, the indirect effect of transformational leadership → employee’s thriving at work → innovative behavior was also significant at the 95% confidence level with a value of *β* = 0.0254 (LLCI = 0.0065, ULCI = 0.0494). These results further confirm H2 and H3.

**Table 3:** Sequential mediation effect (PROCESS macro model 6 analysis).

B	SE	*t*	*P*	LLCI	ULCI
Total effect of transformational leadership on innovative behavior					
0.2840	0.0388	7.3191	0.0000	0.2077	0.3374
Direct effect of transformational leadership on innovative behavior					
0.1303	0.0350	3.7218	0.0002	0.0615	0.1992

	B	SE	LLCI	ULCI
Indirect effect of transformational leadership on innovative behavior				
Total	0.1537	0.0280	0.1001	0.2101
TL—PC—IB	0.0959	0.0251	0.0525	0.1497
TL—TW—IB	0.0254	0.0110	0.0065	0.0494
TL—PC—TW—IB	0.0324	0.0126	0.0095	0.0582

Boot LLCI: Lower limit within 95% confidence interval of Bootstrap indirect effect.

Boot ULCI: Upper limit within 95% confidence interval of Bootstrap indirect effect.

The indirect effect of the sequential mediation of transformational leadership → psychological capital → thriving at work → innovative behavior was significant at the 95% confidence level, with a value of *β* = 0.0324 (LLCI = 0.0095, ULCI = 0.0582). Additionally, the total indirect effect of the entire model was significant at the 95% confidence level, with a value of *β* = 0.1303 (LLCI = 0.0615, ULCI = 0.1992). These results support hypothesis 4, indicating that psychological capital and thriving at work sequentially mediate the relationship between transformational leadership and innovative behavior.

The total effect of transformational leadership on innovative behavior was also significant (*β* = 0.2840, *p* < 0.001, 95% CI [0.2077, 0.3374]). As the confidence interval coefficients do not include zero, the overall model in this study is considered statistically significant.

## 5 Discussion

### 5.1 Theoretical implications

The theoretical implications of this study are as follows. First, it enhances the understanding of how transformational leadership in IT firms influences employees’ innovative behavior. While Opland et al. (2022) emphasized the need to investigate the role of digital tools as a point of interaction between leaders and employees in driving innovation, this study found that digital tools as environmental factors did not significantly impact employees’ innovative behavior.

Instead, drawing from the conservation of resources theory, the findings suggest that a leader’s strong vision and personal attention play a more critical role than the use of digital tools. Specifically, the emotional resources and intellectual stimulation provided by leaders facilitate innovation that extends beyond individual growth, highlighting the importance of leadership qualities in fostering innovation.

Second, this study provides a deeper understanding of the psychological conditions underlying the relationship between transformational leadership and innovative behavior in IT firms. Although psychological capital is a significant driver of positive individual behavior, it has not been thoroughly explored in this context (Luthans et al., 2013; Vilarino del Castillo and Lopez-Zafra, 2022).

This study extends the existing literature by highlighting the importance of transformational leadership in enhancing employees’ psychological capital within the IT industry. It also demonstrates how psychological capital fosters innovative behavior by shaping resources and cognitive processes to address job challenges and achieve goals (Ren and Sun, 2023).

Third, thriving at work equips IT professionals with the psychological resources necessary to solve complex problems and transform innovative ideas into reality. Spreitzer et al. (2005) emphasized the importance of investigating how work unit environments, resources, and agentic work behavior influence employees’ thriving at work. This is particularly relevant as transformational leadership emotionally energizes employees to perform at consistently high levels, thereby fostering innovative behavior (Shahid and Muchiri, 2019). Furthermore, social cognitive theory reinforces the notion that transformational leadership reduces the psychological risks associated with uncertainty. It enables employees to develop flexible cognitive processes and sustain positive energy, both of which contribute to innovative behavior (Khan, 2023; Men, 2014).

Fourth, examining the relationship between transformational leadership and innovative behavior in isolation may hinder a full understanding of its complexity. To provide a more comprehensive perspective, this study explores the dual mediating effects of psychological capital and thriving at work as mechanisms influencing employees’ psychological states. The findings indicate that transformational leadership can inspire spontaneous and constructive behavior through employees’ positive psychological states and thriving at work.

This study extends previous research by illustrating how transformational leadership addresses employees’ psychological needs, fostering innovative behavior through the lens of the caravan of resources perspective and social cognitive theory (Yin et al., 2020; Hobfoll et al., 2018; Hildenbrand et al., 2018).

### 5.2 Practical implications

The findings of this study offer several practical implications.

First, leaders in technology-intensive industries should enhance task interdependence among employees to facilitate the resolution of technical problems and create an environment where tacit knowledge can be codified. Leaders must support employees in maximizing their skills and knowledge, remain open to new ideas, and provide opportunities for employees to explore and implement those ideas.

Leaders should ensure easy access to necessary resources and offer intellectual stimulation to encourage diverse perspectives in problem-solving. Actively promoting experimentation with new ideas is also critical. Furthermore, leaders should establish structures and a culture that align organizational goals with team collaboration. This includes enabling employees to participate in work-related discussions and activities at any time, fostering alignment and cooperation among team members.

Second, instead of overly relying on the latest digital tools or generational traits, IT companies should prioritize strengthening their employees’ psychological well-being. Specifically, leaders should be encouraged to focus on the psychological state of their employees when providing motivation and feedback, as this is a key factor influencing innovative behavior. In the face of rapid technological advancements and increasing complexity, leaders must clearly communicate the goals and significance of projects, reinforcing the relevance of employees’ work to the organization’s objectives.

Leaders should focus on enhancing employees’ psychological capital by fostering supportive relationships and providing the necessary resources to realize ideas promptly. Additionally, leaders should act as role models, demonstrating clear expectations and a passion for innovation, thereby instilling confidence in team members that they can achieve these goals.

Third, leaders should focus on encouraging employees to remain energized and optimistic at work while fostering continuous learning. By cultivating a culture of innovation, leaders can create an environment where skill-diverse employees are empowered to reimagine their work, derive greater meaning, and experience a sense of thriving at work.

Psychological capital and thriving at work play crucial roles in helping employees view challenges positively and recognize their significance within the organization when learning and applying new skills. These factors can also guide leaders in assessing how effectively they have integrated employee psychology into their workplace strategies to foster innovative behavior.

### 5.3 Limitation and directions for future research

Although this study explored the impact of transformational leadership on innovative behavior and highlighted the roles of employees’ psychological capital and thriving at work in this process, it has some limitations.

First, all variables in this study were self-reported, which may introduce to common method bias (Podsakoff et al., 2003). To address these concerns, Harman’s single-factor test was conducted, revealing a total explained variance of 29.758%. This result suggests that the likelihood of result distortion due to common method bias is relatively low. Future research should consider employing paired studies involving both leaders and employees to minimize such bias.

Second, the findings of this study are based on cross-sectional data collected at a single point in time. Korea has distinctive environmental characteristics, including high digital literacy and rapid technology adoption rates, which are likely to serve as significant contextual variables influencing how IT tools impact innovation and psychological capital. Future research would benefit from comparative studies across diverse cultural contexts to better understand these relationships. Additionally, to establish stronger causal relationships between variables, future studies should adopt longitudinal designs to track changes over time.

Moreover, as this study focused exclusively on the IT industry—where innovative behavior and intellectual assets are particularly critical—there is a risk of generalization error. Different industries operate with varying products and resources, which may influence the role and effectiveness of digital tools (Sun et al., 2024). Future research should expand the investigation of the causal relationship between transformational leadership and the use of digital tools by conducting comparative studies across different industries and employee groups.

This study focused solely on the psychological mechanisms underlying the relationship between transformational leadership and employees’ innovative behavior, without considering potential moderating variables that might influence this relationship. Factors such as organizational culture, work environment, and individual characteristics could interact with transformational leadership to more effectively foster innovative behavior. Future research should explore these relationships by incorporating a variety of moderating variables.

Additionally, this study examined the relationship between transformational leadership and innovative behavior at the individual level. However, it is equally important to investigate group-level variables, such as teamwork and hierarchical relationships. Understanding how new ideas and approaches emerge among team members could provide valuable insights into addressing human resource challenges. Future studies should deepen our understanding of the potential drivers of transformational leadership and innovative behavior at the group level (Khan et al., 2024; Bednall et al., 2018).

This study was unable to determine the precise causal relationship between demands and resources in transformational leadership and innovative behavior. Bauer et al. (2014) found that the balance between resources and demands influences psychological capital. This suggests that individuals with greater psychological stability are better equipped to utilize the resources necessary for innovative behavior, thereby mitigating negative demands. It is crucial to examine how employees perceive transformational leadership and behavior and to assess the resulting positive and negative impacts.

Although this study demonstrated that psychological capital and thriving at work act as dual mediators between transformational leadership and innovative behavior, it is possible that additional psychological variables may also play a role. For instance, motivation could significantly influence the pathway to innovative behavior, with factors such as team climate, psychological empowerment, and knowledge sharing serving as potential contributors. Further research is required to incorporate a broader range of psychological variables to deepen our understanding of these relationships.

Finally, Hayes et al., 2010 argued that the causal relationships among the parameters in a serial mediation model should be theoretically validated through prior research. However, two variables can be challenging in cross-sectional surveys collected at a single point in time. This limitation makes it difficult to isolate and analyze the independent effects of the variables. To address this issue, future research should explore the parallel mediating effects of leaders’ transformational behavior on innovative behavior through employees’ psychological capital and thriving at work.

## Data Availability

The original contributions presented in the study are included in the article/supplementary material, further inquiries can be directed to the corresponding author.
